# Heartbeat Induces a Cortical Theta-Synchronized Network in the Resting State

**DOI:** 10.1523/ENEURO.0200-19.2019

**Published:** 2019-08-08

**Authors:** Jaejoong Kim, Bumseok Jeong

**Affiliations:** 1 Graduate School of Medical Science and Engineering, Korea Advanced Institute for Science and Technology (KAIST), Daejeon 34141, Republic of Korea; 2 KI for Health Science and Technology, KAIST Institute, KAIST, Daejeon 34141, Republic of Korea

**Keywords:** emotion, heartbeat-induced network, interoception, MEG, resting state network

## Abstract

In the resting state, heartbeats evoke cortical responses called heartbeat-evoked responses (HERs), which reflect cortical cardiac interoceptive processing. While previous studies have reported that the heartbeat evokes cortical responses at a regional level, whether the heartbeat induces synchronization between regions to form a network structure remains unknown. Using resting-state MEG data from 85 human subjects of both genders, we first showed that heartbeat increases the phase synchronization between cortical regions in the theta frequency but not in other frequency bands. This increase in synchronization between cortical regions formed a network structure called the heartbeat-induced network (HIN), which did not reflect artificial phase synchronization. In the HIN, the left inferior temporal gyrus and parahippocampal gyrus played a central role as hubs. Furthermore, the HIN was modularized, containing five subnetworks called modules. In particular, module 1 played a central role in between-module interactions in the HIN. Furthermore, synchronization within module 1 had a positive association with the mood of an individual. In this study, we show the existence of the HIN and its network properties, advancing the current understanding of cortical heartbeat processing and its relationship with mood, which was previously confined to region level.

## Significance Statement

Complex brain processing usually occurs at a network level, which requires an interaction between brain regions. However, despite its importance in homeostasis and affective processing, a network level processing of cardiac interoception has not been investigated. Here, we first provided an evidence that the heartbeat induces phase synchronizations between cortical regions those comprise a heartbeat-induced network (HIN) with control analyses excluding the possibility of an artificial synchronization. Furthermore, by applying graph-theoretical analysis, we find hubs of the HIN and found out that it is a modularized network with five modules. Finally, we also showed the relationship between the participants’ mood and the HIN. These results provide the first evidence of network-level heartbeat processing and its relevance with emotion.

## Introduction

One of the important purposes of the brain is to maintain homeostasis by continuously sensing the homeostatic state (for example, visceral sensations and immunologic signals), which is termed interoception, and the brain regulates the bodily condition using this homeostatic information even in the resting state (Craig, 2009). Recently, interoceptive processing, especially at the cortical level, has been proposed to play various roles not only in reflective homeostatic regulation but also in psychological processes, including affective and cognitive processing (Tsakiris and De Preester, 2018). Therefore, understanding the precise mechanism of cortical interoceptive processing is important for understanding “embodied” emotion and cognition.

Because the brain processes interoceptive information, there exist brain responses related to visceral signal processing. For example, the heartbeat signal evokes cortical activity defined as heartbeat-evoked responses (HERs; Pollatos and Schandry, 2004). HERs are associated with many psychological processes, including heartbeat awareness (Pollatos and Schandry, 2004), emotion processing (Couto et al., 2015; Maister et al., 2017; Kim et al., 2019), visual awareness (Park et al., 2014), bodily self-consciousness (Park et al., 2016), and autobiographical self-related processing (Babo-Rebelo et al., 2016a,b). Furthermore, disruption of resting-state HERs is known to be related to emotion-related psychiatric diseases/disorders, such as depression and borderline personality disorder (Terhaar et al., 2012; Müller et al., 2015).

While previous studies have shown that the heartbeat evokes region-level or sensor-level cortical responses and is related to a variety of psychological states and functions, whether the heartbeat also induces interactions between cortical regions remains unknown. However, previous fMRI studies have shown that network-level fluctuations vary with visceral signal-related measures such as heart rate variability (HRV; Chang et al., 2013; Rebollo et al., 2018), indicating that heartbeat-related interactions may occur between cortical regions. Therefore, we hypothesized that the heartbeat induces functional coupling between cortical regions involved in heartbeat processing and forms a network structure in the resting state. In this study, using a resting-state MEG dataset, we investigated the heartbeat-induced network (HIN), which was defined as a network composed of significantly increased phase synchronization between regions compared with baseline values. We first showed the existence of the HIN and that it does not reflect artificial synchronization between cortical regions. Then, we investigated the properties of the HIN using graph-theoretical measures. In particular, we first investigated the hubs of the HIN, which play a central role in the HIN. Next, we investigated the modularized property of the HIN to determine whether the HIN is one homogeneous network or can be segregated into several subnetworks. Finally, cardiac interoceptive processing has been suggested to be closely related to an affective state of an individual (Terhaar et al., 2012; Müller et al., 2015). Therefore, we hypothesized that synchronization within the HIN, reflecting network-level cardiac interoceptive processing, is related to an affective state of an individual. We tested this hypothesis by investigating the relationship between synchronization within the HIN and affective state scores of study participants.

## Materials and Methods

### Dataset description

Resting-state MEG data from 89 subjects collected from the Human Connectome Project (HCP) S1200 data release were used in this study (Larson-Prior et al., 2013; Van Essen et al., 2013, RRID: SCR_008749). All subjects were young (22–35 years of age) and healthy. MEG recordings were collected on a whole-head Magnes 3600 scanner (4D Neuroimaging) with 248 magnetometer channels at a sampling rate of 2034.51 Hz. Recordings were performed in three sessions, and each session lasted 6 min. HERs were extracted from the preprocessed version of the MEG dataset, which is publicly available at Connectome DB (Hodge et al., 2016). The preprocessing pipeline of HCP data included segmentation of the raw data into epochs of 2 s and removal of bad segments and bad channels. Importantly, an independent component analysis (ICA; Hämäläinen et al., 1993) was applied to remove cardiac field artifacts (CFAs) and electrooculography (EOG)-related artifacts, and the data were finally downsampled to 508.68 Hz. Notably, because these preprocessed data did not include electrocardiogram (ECG) recordings, which are essential for HER extraction, we used an ECG recording included in the raw MEG data. Among 89 subjects, four subjects were excluded (IDs 133019, 140117, 149741, and 17746); we failed to detect the R-peak in the ECG recording of subject 149741, and in subjects 133019, 140117, and 177741, an excessive error occurred when performing Brainnetome atlas-based (Fan et al., 2016) source time course extraction [the time course extraction of >16 cortical regions (among 210 cortical regions) failed in this subject]. Finally, 85 subjects were included in our analysis (47 males and 38 females).

### MEG analysis

#### HER extraction procedure

HER extraction and preprocessing were performed using the FieldTrip toolbox (Oostenveld et al., 2011, RRID: SCR_004849). First, preprocessed HCP MEG data, which were initially segmented into 2-s epochs, were concatenated into one continuous time series such that each segment was realigned to its original location in the raw MEG recording (preprocessed data contained information about the locations of each segment in the raw MEG recording). Because bad segments were removed, the concatenated continuous time series data had empty spaces where the bad segments existed. These empty spaces were replaced by NaN. Next, the R-peak was detected in the ECG recordings using the Pan-Tomkins algorithm (Pan and Tompkins, 1985). Then, epoching of HERs from 900 ms before the R-peak to 1800 ms after the R-peak was performed on the concatenated continuous MEG data. As mentioned above, the concatenated MEG data contained the time window including NaN; thus, the HER epoching procedure resulted in some NaN-containing epochs. These NaN-containing HER epochs were removed. Finally, the HER extraction procedure resulted in 840.8 (±138.7) HER epochs on average. The mean interbeat interval (IBI) of every subject was 981.8 ms [±151.5, corresponding heart rate: 61.11 beats per minute (BPM)], with a range of 675.4–1374.2 ms (heart rate range: 43.67–88.84 BPM).

#### Source reconstruction of HERs

All sensor HER data were source-reconstructed using the linearly constrained minimum variance (LCMV) beamformer methods (Van Veen et al., 1997) provided in the FieldTrip toolbox in a manner similar to that used in a previous study (Heusser et al., 2016). A common spatial filter was estimated for each source point using HER data from all trials, an HCP-provided single-shell volume conduction head model and an HCP-provided 4-mm grid source model for every subject (Larson-Prior et al., 2013). Then, this common spatial filter was applied to sensor HER data (sensor * time matrices) to calculate the time courses of each source. Finally, we used Brainnetome atlas-based parcellation (Fan et al., 2016, RRID: SCR_014091) to perform a region of interest (ROI)-based connectivity analysis. Among the total of 246 brain regions, because an HER is known to mainly reflect cortical heartbeat processing (Pollatos et al., 2005) and the deep source activity estimation including the subcortex and cerebellum in MEG data are less reliable than cortical source estimation (Hämäläinen et al., 1993), we excluded 36 subcortical cerebellar regions. We also excluded 15 cortical regions among the remaining 210 cortical regions because the source reconstruction procedure failed to extract time courses in these regions in at least one subject (indicating that these regions did not contain source vertices in some participants). Therefore, the time courses of vertices within each of the 195 cortical regions (Extended Data Table 1-1) were averaged. This final step produced the time courses of HERs for all 195 cortical regions, epochs and subjects.

10.1523/ENEURO.0200-19.2019.t1-1 Extended Data Table 1-1 List of 195 cortical regions used in the analysis and the graph theoretical properties of each region Download Table 1-1, DOCX file.

#### Calculation of the debiased wPLI estimator in the theta frequency range between cortical regions

The debiased estimator for weighted phase lag index (wPLI-D; Vinck et al., 2011) was used as a measure of functional connectivity between cortical regions. The weighted phase lag index (wPLI; Vinck et al., 2011) is a measure of phase coherence and is robust to the spurious connectivity induced by volume conduction, which is reflected in “zero-phase synchronization” between sources. Furthermore, the wPLI is invariant to linear mixing of two dependent sources (Vinck et al., 2011; Palva et al., 2018), and in the presence of true interactions, this measure is immune to false-positive detection (Palva et al., 2018). Because a direct estimator of the wPLI is biased by sample size (Vinck et al., 2011), we used the debiased wPLI estimator (wPLI-D; Vinck et al., 2011), which ranges from zero to one (maximum coherence). We hypothesized that synchronization would occur in the theta band (4–7 Hz), which is the frequency band with the strongest increase in phase synchronization within regions according to a previous study (Park et al., 2018). First, complex Morlet wavelet transformation was performed on every trial (which was epoched from –900 to 1800 ms R-peak) with a 20-ms time step starting from –300 to 600 ms R-peak and a frequency ranging from 4–7 Hz with 1-Hz steps. Four cycles were used in the wavelet transformation procedure. Then, the wPLI-D was calculated for every pair of regions in each time and frequency step. wPLI-Ds from 4 to 7 Hz were averaged to obtain the wPLI-D of the theta frequency range. These procedures resulted in 195 (number of ROIs) by 195 by 31 (time windows from –300 to 600 ms at the R-peak with 20-ms steps) wPLI-D matrices for each subject. Additionally, although our frequency band of interest was the theta band, which has been shown to be a major frequency band with respect to HERs, we tested whether similar HINs exist in the α (8–13 Hz) and β (14–29 Hz) bands by using the same pipeline used in the theta band HIN.

#### Identification of the HIN using network-based statistics (NBSs)

We compared the wPLI-Ds between the baseline period, which was defined as a time window 300–100 ms before the R-peak onset, and a time window 200–600 ms after the R-peak onset, which is the time window in which the effects of HERs were reported in most previous HER studies (Fukushima et al., 2011; Pollatos and Schandry, 2004), to determine whether the heartbeat induced a network composed of significantly increased phase synchronization between regions, and we called this time window the “induced” period. The 200-ms period after the R-peak onset corresponds to the approximate time that the heartbeat signal enters the CNS following carotid baroreceptor stimulation, which is the major input path of the heartbeat to the CNS (Eckberg and Sleight, 1992). However, note that because the heartbeat signal is conveyed to the CNS via various pathways except this pathway including somatosensory pathway via spinal cord or stimulation of cardiac afferent neuron at heart wall, the timing of this CNS entrance of the heartbeat signal could be varying (Park and Blanke, 2019). The baseline period used in the present study is the same period used in a previous study of HER-induced phase synchronization within regions. This baseline period was postulated to avoid cardiac artifacts around the ECG P-wave (Park et al., 2018).

We then performed a group-level NBS (Zalesky et al., 2010; RRID:SCR_002454) analysis, which is a statistical method that controls multiple comparisons at the network level. This analysis enabled us to identify a network composed of significantly increased wPLI-Ds between cortical regions in the induced period compared to the baseline period at the group level. First, baseline and induced wPLI-D matrices were computed by averaging wPLI-Ds from each time window for every subject, which resulted in one baseline wPLI-D matrix and one induced wPLI-D matrix for each subject. Second, multiple paired *t* tests comparing wPLI-Ds from the induced period and the baseline wPLI-D were performed for every pair of cortical regions, which resulted in one matrix of *t* values from these paired *t* tests. Then, a threshold *t* value of 2.51 was applied to the matrix of *t* values, and a *t* value < 2.51 was therefore set to 0. The network statistic was computed by adding the *t* values of all the connected components in the thresholded matrix (a connected component refers to any two nodes within a component are connected by a path of edges). Next, a null distribution of the network statistic was created from 5000 permutations by randomly permuting an element of the induced wPLI-D matrices and the baseline wPLI-D matrices within each subject. Finally, network-level familywise-error (FWE)-corrected *p* values of the network were obtained using the original network statistic and null distribution. Next, we constructed a heartbeat-induced synchronization (HIS) matrix whose elements corresponded to the increase in the wPLI-D in the induced period compared to that in the baseline period, and each element was significant in the NBS results. Therefore, the HIS matrix was composed of elements with significantly increased wPLI-Ds in the group-level NBS analysis and represents the structure of the HIN.

#### Examination of increased theta phase synchronization between ECG signals and brain regions

We postulated that the HIN that we identified may represent an artificial increase in phase synchronization caused by a CFAs. We expected that if an electromagnetic field induced by cardiac contractile activity directly influenced both regions A and B and this effect artificially increased phase synchronization between these two regions, then phase synchronization would increase between regions A and B, and the phase synchronization between the ECG signal and both regions A and B should increase after a heartbeat because the same electromagnetic field induced by cardiac contraction influenced all three signals, including the ECG signal and the signals from regions A and B. We assessed whether theta-phase synchronization between ECG signals and brain regions increased during an induced period (200–600 ms after the R-peak) compared to that in the baseline period to test this hypothesis. We calculated the wPLI-Ds between ECG signals and 195 cortical regions in the theta band for every subject, which resulted in two 195 by one vector of ECG-brain region wPLI-Ds from the baseline and induced periods for every subject. Then, we performed 195 group-level paired *t* tests between the wPLI-Ds from the induced and baseline periods for all 195 cortical regions to determine which wPLI-Ds between each ECG-brain region pair were significantly increased in the induced period compared to that at baseline.

#### The forward and inverse-modeled trial-shuffled surrogate method for evoked component estimation of the HIN

We next tested whether phase synchronization in the HIN reflected artificial synchronization due to evoked responses within distributed regions, which are phase-locked to the heartbeat (Hirvonen et al., 2018), using the forward and inverse-modeled trial-shuffled surrogate method, which was used to identify an evoked component of phase synchronization in a previous study (Hirvonen et al., 2018). This method selectively and more effectively identifies true-induced interareal interactions compared to the conventional trial shuffling method (Lachaux et al., 1999). Briefly, using the source-modeled single-trial data, the time course of each source vertex within a particular region was simulated with the region time courses of a random trial (trial shuffle) and using the forward model, sensor-level surrogate data were generated. Finally, the sensor-level surrogate data were source-reconstructed, and the wPLI-Ds between cortical regions were calculated with procedures identical to those used for the real data. As noted in a previous study (Hirvonen et al., 2018), by using this procedure, surrogate data contain both evoked, stimulus-phase-locked components and signal spread caused by MEG data acquisition and inverse modeling, while non-stimulus locked (induced) phase synchronization between regions is eliminated. Therefore, by comparing the HIN of the surrogate data with the HIN of the real data, we can identify whether phase synchronization within the HIN was caused by evoked responses. We established 20 sets of surrogate data and compared phase synchronization within the HIN between the surrogate data and the real data.

#### Network properties of the HIN

After confirming that the HIN does not reflect artificial synchronization induced by either CFAs or evoked responses, we identified the following characteristics of the HIN. First, we identified the hubs of the HIN, which play an important role in connecting regions within the HIN. Then, we identified whether the HIN is one homogeneous network or can be divided into subnetworks called modules, indicating that the HIN is modularized. Additionally, we also investigated how synchronization within the HIN changes over time by summing the wPLI-D of every HIN edge at every time point from –300 to 600 ms.

#### Identification of the hubs of the HIN

One of the important features of a network is the hub of the network, which is defined as a node that plays an important role within a network, such as connecting nodes (Fornito et al., 2016). To identify the hubs of the HIN, we calculated the strength and betweenness centrality of each region. The strength of a node is defined as a sum of the weights of all edges connected to that node, and betweenness centrality is defined as the fraction of all the shortest paths in a network that pass through a given node (Brandes, 2001). The graph-theoretical measures used to define hubs were calculated using the functions of the brain connectivity toolbox (BCT; Rubinov and Sporns, 2010; RRID: SCR_004841).

#### Identification of the modularized structure of the HIN

Because we expected that the HIN would have a modular structure, we applied a community detection algorithm to the HIS matrix to determine how the HIN is partitioned into different subnetworks. However, to identify whether the HIN is modularized, one should examine the extent of modularity compared to random networks. Therefore, we compared the “modularity index” of the HIN with 100 random networks (Bassett et al., 2011). Optimal partitioning of cortical regions was performed using the Louvain greedy algorithm (Blondel et al., 2008) to maximize the modularity index Q formulated using the following equation:

$$\text{Q }=\frac{1}{2\mu }\underset{ij}{\sum }[A_{ij}-\text{γ}P_{ij}]\delta (g_{i},g_{j}).$$

In this equation, $A_{ij}$ represents the strength of the edge between node i and node j, $P_{ij}$ represents the expected weight between node i and node j, μ is the sum of the strengths of all edges in the network, and $\delta (g_{i}g_{j})$ is 1 if node i and j belong to the same community and 0 otherwise ($g_{i}$ is a label of the community to which node i belongs). The resolution parameter γ was set to 1, which is a default value. However, because the partition that maximizes Q can vary across each algorithm run, we used the consensus partition method to identify the most representative partition S (Lancichinetti and Fortunato, 2012) using the functions of the BCT (Rubinov and Sporns, 2010). The consensus partition procedure, which is identical to a previously reported procedure (Fornito et al., 2016), is briefly explained below. First, a community detection algorithm (Louvain greedy algorithm) was run 10,000 times to create 10,000 partitions. Second, the agreement matrix D was constructed. Each element of D corresponded to the proportion of the number of times that nodes i and j were in the same module to the number of total iterations. Third, a threshold τ = 0.2 was applied to D. The value of τ was set to <0.4 as recommended in a previous study (Lancichinetti and Fortunato, 2012). Fourth, community detection was performed 10,000 times using D, which created another agreement matrix, D’. Fifth, steps 2 through 4 were repeated until the consensus matrix exhibited a block-diagonal structure in which all edge weights equaled one for node pairs in the same community and zero otherwise. We initially constructed the agreement matrix with 10,000 iterations of the HIS matrix. Then, 10,000 partitions were provided as the functional input for steps 2 through 4, and these processes were repeated until convergence was achieved. By this consensus partitioning procedure, we achieve optimal partitioning and obtain the modularity index Q of this optimal partition. To test whether the HIN is modularized, we constructed 100 random networks with preservation of the weight distribution and then applied the same consensus partition procedure on these random networks. As a result, we obtained 100 Q values of each random network, which constituted a surrogate distribution of the Q. Then, we tested the location of the Q value of the HIN in this surrogate distribution.

#### Properties of each module of the HIN

After partitioning the HIN, we identified the properties of each module of the HIN. Specifically, we extracted the time course of within-module synchronization for each module, which was defined as the sum of the edge weights within each module at every time point, and then determined which node was the “connector hub” of the modules. These connector hubs connect modules and enable effective interactions between modules and are defined by graph-theoretical measurements called the within-module degree *z* score (Guimera and Amaral, 2005) and the participation coefficient (Guimera and Amaral, 2005). The within-module degree *z* score quantifies the normalized within-module strength, while the participation coefficient quantifies a node’s participation in each module. Using within-module degree *z* scores and participation coefficients, we defined the role of every node according to the *z*-P classification (Guimera and Amaral, 2005). In particular, the connector hub is a node with many connections within the module to which the node belongs and also forms many connections with nodes of other modules; thus, the connector hub efficiently connects nodes within one module to nodes of other modules (Fornito et al., 2016). In our study, the connector hub was defined as a node with a within-module degree *z* score >2.5 and a participation coefficient (P) > 0.3 (Guimera and Amaral, 2005; Fornito et al., 2016).

#### Identification of between-module interactions using graph-theoretical analysis

We next investigated synchronization patterns between modules. Between-module synchronization of _5_C_2_ pairs of modules was computed by the sum of the edge weights between each module, which resulted in a 5 × 5 between-module synchronization matrix. By applying graph-theoretical analysis to this between-module synchronization matrix, we identified which module plays a central role within the HIN using module-level nodal strength and betweenness centrality.

#### The relationship between emotional status and the HIN

To identify relationships between the HIN and participants’ emotional states, we used the emotional statuses included in the HCP data. The HCP data included a measure for six negative emotional affective states, including anger-affect, anger-hostility, anger-aggression, fear-affect, fear-somatic, and sadness, and positive affect surveys were retrieved from the NIH Toolbox (Gershon et al., 2010). To reduce dimensions, we performed a principal component analysis of seven survey scores and extracted the first principal component (PC) reflecting the moods of the participants. Then, we fit a stepwise linear regression model in which the first PC was a dependent variable to the within-module and between-module synchronizations of the five HIN modules.

## Results

### Theta-phase synchronization between cortical regions increased after the heartbeat, confirming the existence of the HIN

The NBS analysis showed a network displaying a significant increase in phase synchronization in the induced period compared to that in the baseline period (network-level FWE-corrected *p* < 0.001), revealing the existence of the HIN. The density of the network was 9.2%, indicating that among the total of _195_C_2_ pairs of regions, 9.2% of the region pairs showed significantly increased phase synchronization after the heartbeat. Additionally, no HIN formed in the α-frequency or β-frequency bands.

### No significant change in phase synchronization occurred between ECG signals and cortical regions

**Figure 1.**
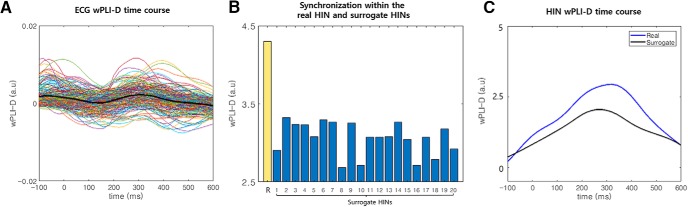
Results of the control analysis. ***A***, The time course of synchronization between the ECG signal and HIN regions. We plotted the wPLI-D in the theta band between the ECG signal and HIN regions for all HIN regions (thin colored lines). In addition, the averaged synchronization time course was also plotted (thick black line). These time courses show similar levels of synchronization between the baseline and induced periods. ***B***, Comparison of the synchronization within the HIN between real and surrogate data in the induced period. We generated 20 surrogate datasets without an induced component of synchronization and compared synchronization within the HIN between real (yellow bar with the label R) and surrogate data (blue bar). This figure shows that the synchronization within the real data are stronger than the synchronization in all 20 surrogate datasets in the induced period, indicating that the synchronization within the HIN cannot be explained by artificial synchronization caused by evoked responses (Extended Data Fig. 1-1). ***C***, The time courses of synchronization within the HIN for real and surrogate data. This panel shows that the synchronization within the real data are stronger than the mean synchronization in the surrogate datasets in the induced period (Extended Data Fig. 1-1). Note that in ***A***, ***B***, baseline subtraction was performed (–300 to –100 ms at the R-peak).

The paired *t* tests (wPLI-Ds for ECG signals and cortical regions) comparing responses between the induced period (200–600 ms after the R-peak) and the baseline period did not reveal a significant increase or decrease in wPLI-Ds between ECG signals and cortical regions in the theta band (the minimum *p* value among the 195 cortical regions was *p* = 0.104 [false discovery rate (FDR)-corrected] with *t*_(86)_ = 2.97 in the “right postcentral gyrus A2”; Fig. 1*A*). If the electromagnetic field generated by cardiac contractile activity induced artificially increased phase synchronization between regions in the HIN compared with the baseline period, then the ECG signal originating from the same electromagnetic field should show increased phase synchronization with cortical regions within the HIN. However, phase synchronization between cortical regions and ECG signals did not change in the induced period compared to baseline, while phase synchronization between the regions in the HIN increased in the induced period, indicating that the increased theta-phase synchronization between cortical regions in the HIN was not caused by CFAs (Fig. 1*A*). Furthermore, the HIN was not likely caused by a pulse artifact (PA), which occurs when sensors are influenced (moved) by vascular pulsation. In our study, we used MEG data, and MEG sensors do not directly contact the subject; thus, a vessel cannot induce pulsatile movement of the sensors to cause a PA. To our knowledge, no previous studies have reported a PA in MEG recordings. Furthermore, according to a previous HER study using electrocorticography (ECoG; Kern et al., 2013), if PA-induced artificial synchrony occurs between ECoG electrodes, then the ECG and ECoG electrodes likely display high phase synchronization (Kern et al., 2013), which was not observed in our results. By summarizing these results, the HIN that we identified in the theta frequency band was not caused by an artificial increase in phase synchronization induced by CFAs or a PA. While the theta-phase synchronization between ECG signals and cortical regions was not increased compared to baseline, a CFA-induced increase in phase synchronization compared with baseline may exist in lower frequency bands, such as the δ band (0.5–4 Hz), because cardiac contractile activity typically occurs at a rate of 60–100 BPM, which corresponds to a frequency of 1–1.67 Hz belonging to the δ band. Similarly, in our data, the subjects displayed a maximum heart rate of 88.84 BPM (∼ 1.48 Hz); therefore, the CFAs or PA induced by pulsation may have increased artificial synchronization in the δ band.

10.1523/ENEURO.0200-19.2019.f1-1 Extended Data Figure 1-1 wPLI-D in the induced and baseline period for real and surrogate data. This figure shows the wPLI-D in the induced (blue) and baseline (orange) period separately. R represents the wPLI-D of the real data and others represents wPLI-D of 20 surrogate data. One can notice that the synchronization within the HIN is much stronger in the real data for both induced and baseline period. Note that, an increase of induced synchronization was also strongest in the real data compared to the surrogate data (Fig. 1*B*). Download Figure 1-1, PDF file.

### The HIN is not composed of artificially increased synchronization induced by evoked responses

We compared the synchronization within the HIN in 20 surrogate datasets and in the real data. The results showed that the HIS within the HIN in the real data were significantly stronger than that in all 20 surrogate datasets (Monte Carlo *p* < 0.05; Fig. 1*B*,*C*; Extended Data Fig. 1-1), indicating that the synchronization within the HIN could not be explained by artificial synchronization caused by evoked responses in distributed regions. Notably, if the HIN is composed of evoked responses in distributed regions, the existence of only a small proportion of edges among all possible edges (9.2%) within HIN regions is unlikely.

### Network properties of the HIN

**Figure 2.**
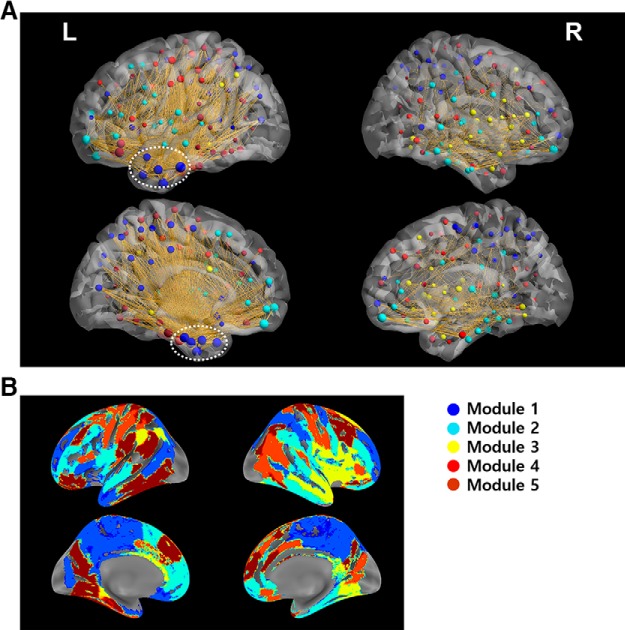
Structures of the HIN. ***A***, Synchronization patterns within the HIN. The figure shows that the synchronization within the HIN is concentrated in the left inferior temporal region (white dashed circles), particularly in the polar part and the parahippocampal gyrus, which are hubs of the HIN. Furthermore, these regions were contained in module 1. ***B***, Spatial pattern of each module of the HIN. In this spatial map of each module, module 1 contained most of the polar part of the left inferior temporal regions and the parahippocampal gyrus. The posteromedial part of the bilateral hemispheres was also contained in this module. Module 2 contained the ventromedial and orbitofrontal cortices, which are also hubs of the HIN.

Within the HIN, left inferior temporal regions including the temporal pole and parahippocampal gyrus had high strength and betweenness centrality (Fig. 2*A*; Table 1). Figure 2*A* shows that the connections between HIN regions are centered at the polar part of an inferior temporal region including the inferior temporal gyrus and parahippocampal gyrus. Specifically, “left inferior temporal gyrus A20il” had the highest betweenness centrality and strength among the regions (Table 1), suggesting its importance as a hub of the HIN. In addition to these regions, orbitofrontal regions also had a high degree and betweenness centrality. Notably, the HIN was left-dominant such that the HIS between regions was substantially stronger within left hemispheric regions than that within right hemispheric regions (*t*_(84)_ = 5.22, *p* < 0.001 in a paired *t* test comparing induced synchronization between the right and left hemispheres; Fig. 2*A*). Finally, the time course of the synchronization within the HIN showed that the degree of synchronization increases from baseline and was maximal at ∼300 ms after the R-peak (Fig. 1*C*).

**Table 1.** High-strength nodes of the HIN

Region name	BC	Strength	Module
Left inferior temporal gyrus A20il, intermediate lateral area 20	8661	30.1	1
Left parahippocampal gyrus A35/36r, rostral area 35/36	4775	25.9	1
Left inferior temporal gyrus A20iv, intermediate ventral area 20	2781	25.4	4
Left inferior temporal gyrus A20r, rostral area 20	4069	24.8	1
Left fusiform gyrus A20rv, rostroventral area 20	1556	24.3	4

Five regions having high strength are reported here with their betweenness centrality and modules they belong to. Full list of regions and their network characteristics are provided in the Extended Data Table 1-1. BC, betweenness centrality.

### The HIN is a modularized network with five subnetworks, and module 1 plays a central role within the HIN

**Figure 3.**
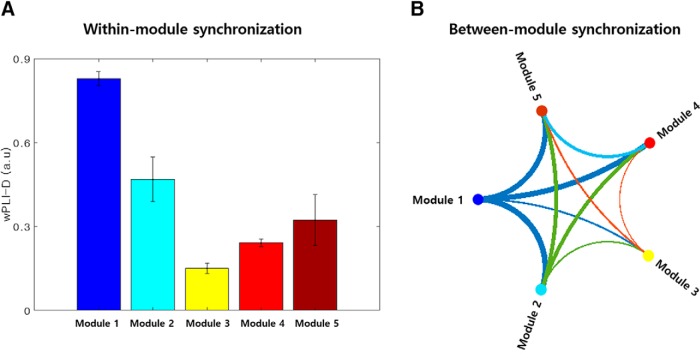
Within-module and between-module synchronization in the HIN. ***A***, Within-module synchronization in the HIN. Within-module synchronization was strongest in module 1, followed by that in module 2. ***B***, Between-module synchronization pattern of the HIN. The between-module synchronization pattern graph shows that module 1 is the center of interaction between modules such that it has strong connections with other modules, which were quantified by the strength of this node.

Based on the consensus partitioning results, the HIN was partitioned into five modules (Fig. 2*A*,*B*). Using the consensus partitioning result for the real data and the consensus partitioning of the random network, we tested whether the HIN is modularized. We found that the HIN had a significantly greater modularity index than the random network (Monte Carlo *p* < 0.01), indicating that the HIN is modularized rather than one homogeneous network. Importantly, among the five modules, the synchronization within module 1 was the strongest (Figs. 2*A*, 3*A*). This module contained most of the polar part of the left inferior temporal gyrus and parahippocampal gyrus (Fig. 2*B*). Specifically, left inferior temporal gyrus A20il, which had the highest betweenness centrality and strength, was also contained in this module. Furthermore, graph-theoretical analysis of the between-module synchronization matrix showed that module 1 was the center of an interaction between the HIN modules with the highest strength (1.29) and betweenness centrality (2) among the modules (Fig. 3*B*). The posteromedial regions including the middle cingulate cortex, supplementary motor area, posterior cingulate and precuneal regions were also contained in this module (Fig. 2*B*). Module 2 had the second strongest within-module synchronization and contained the bilateral ventromedial/orbital frontal regions (Figs. 2*A*, 3*A*). Finally, seven hubs connected each module, most of which were located in the temporal polar regions and orbitofrontal regions.

### The relationship between affective status and synchronization within the HIN

**Figure 4.**
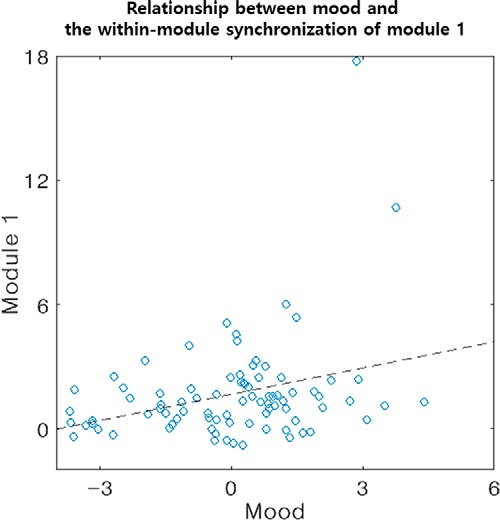
The relationship between mood and the within-module synchronization of module 1. Stepwise linear regression showed that the within-module synchronization of module 1 has a positive association with the moods of the participants.

The first PC explained 47% of the variance of the emotion survey data and had positive loading on the positive affect score and negative loading on the other negative affect scores. Therefore, we surmised that this PC reflected the moods of the participants. Stepwise linear regression analysis resulted in a model that only contained the within-module synchronization of module 1 in the HIN, which explains ∼10% of the variance of the mood data (model *F*_(1,83)_ = 8.95, *p* = 0.004, *R*^2^ = 0.10, β of the within-module synchronization of module 1 = 0.57, *t* = 3.00, *p* = 0.004; Fig. 4), indicating that an individual with higher synchronization within module 1 has a more positive mood or is less likely to experience a negative mood. Notably, the relationship between these two variables was also significant when we applied a robust regression or non-parametric correlation to reduce the effect of outlier points (all *p* < 0.05).

## Discussion

In the resting state, our brain receives cardiac afferent signals, and previous studies have shown regional modulation of HERs. As shown in the present study, we found that the heartbeat induces theta-phase synchronization of cortical regions, thus generating a network that we called the HIN, which did not reflect artificially-induced synchronization. This HIN was not present within other frequency bands, including α and β bands. The synchronization within the HIN was maximal at ∼300 ms after the R-peak, and the left inferior temporal gyrus and parahippocampal gyrus played a central role as hubs. Furthermore, the HIN was a modularized network with five modules. Module 1 included major hubs of the HIN and played a central role in interactions between modules of the HIN. Finally, we found that the stronger synchronization within module 1 of the HIN explained 10% of the variance in mood and had a positive association with mood.

In this study, we first showed that the heartbeat increases “true” induced synchronization between cortical regions by controlling several factors that can cause artificial synchronization. By using the wPLI-D as a synchronization metric, we controlled the effect of zero-phase synchronization caused by volume conduction (Cohen, 2014). Furthermore, we controlled for the possibility of artificially increased synchronization induced by CFAs from cardiac contractile activity or a PA by analyzing the increase in ECG-cortical region phase synchronization. However, because an artifact-induced increase in phase synchronization compared with synchronization at baseline may exist in a lower frequency band, such as the δ band, we suggest that for MEG studies, an investigation of the HIN in the frequency ranges covering the theta and higher frequency bands would be more reliable because these bands are unlikely to be influenced by CFAs or a PA, while an investigation of the HIN in the δ band and lower frequency bands would be less reliable because artifacts and the HIN would be difficult to discriminate in these frequency bands. Lastly, we controlled for large-scale synchronization within the HIN that may occur artificially due to evoked responses in cortical regions. We compared the HIN of real data with that of surrogate data whose non-heartbeat-locked phase relationships were eliminated while evoked components were preserved (Hirvonen et al., 2018) and found that the phase synchronization within the HIN could not be explained by artificial synchronization caused by evoked responses. These results from the control analyses consistently suggest the existence of the HIN, which is likely composed of truly increased neural-phase synchronization induced by the heartbeat.

Several studies have investigated brain regions or networks related to cardiac activity. Chang and colleagues investigated a resting-state brain network that fluctuated with HRV using the dorsal anterior cingulate cortex and amygdala as seed regions (Chang et al., 2013). In the recent reviews of Azzalini et al. (2019), authors mentioned that because HRV was reported to be largely driven by the brain, the HRV-associated resting-state brain networks are likely to be associated with descending influences from the brain to heart (Azzalini et al., 2019). Another recent fMRI study showed regions associated with a low-frequency peripheral pulse fluctuation called an autonomic network (Shokri-Kojori et al., 2018). Based on these studies showing cortical regions or networks related to cardiac activity-related measures, we showed ascending cardiac afferent signal-induced phase synchronization between cortical regions in this study. Furthermore, we quantified the interaction pattern between these regions in the network using graph-theoretical measures, which have not been investigated in previous studies.

In the analysis of the properties of the HIN, synchronization within the HIN was maximal at ∼300 ms after the R-peak, which is the time after the heartbeat enters the CNS (Eckberg and Sleight, 1992). Most of the hubs of the HIN with high strength and betweenness centrality were concentrated around the left inferior temporal gyrus and the parahippocampal gyrus. In particular, the parahippocampal gyrus has been reported to be related to the cardiac cycle duration (Kim et al., 2019) and to be part of an autonomic network (Shokri-Kojori et al., 2018). Additionally, the bilateral orbitofrontal region also had high strength and betweenness centrality, which is a visceromotor region that sends motor signals to the viscera (Kleckner et al., 2017). Notably, unlike previous theories emphasizing the role of the insula in processing interoceptive signals, in our study, the insula did not serve as a hub (Craig, 2009). However, some recent studies of HERs showed that the insula is not always the most important structure for heartbeat processing, but its importance varies according to the task or situation that one is engaged in (Babo-Rebelo et al., 2016a,b; Tsakiris and De Preester, 2018).

Next, we found that the HIN was a modularized network with greater modularity than random networks. The HIN was divided into five modules, and module 1 was the center of interactions between these five modules with high module-level betweenness centrality and strength, indicating that module 1 is responsible for a large portion of the interactions between modules of the HIN. Module 1 contained left inferior temporal regions including the parahippocampal gyrus and posteromedial regions. Interestingly, an autonomic network identified in a previous fMRI study was composed of regions showing stronger interactions with low-frequency peripheral pulse amplitude fluctuations (occurring at ∼0.01–0.09 Hz) than with other brain regions (Shokri-Kojori et al., 2018), and the network had some topological overlap with module 1 such that it also contained a large portion of posteromedial regions and the parahippocampal gyrus. Notably, this previous study also used an HCP dataset (fMRI and behavior data, 18 participants in the previous study were also included in our study) as in our study. Furthermore, while module 1 showed a significant relationship with the mood score, the autonomic network also showed a significant relationship with the emotion PC extracted from the HCP behavioral data of emotion (which is slightly different from our study because the authors not only extracted an emotion PC from the score related to mood but also included other measures such as an emotion recognition score; Shokri-Kojori et al., 2018). The topological overlap between module 1 and the autonomic network, which is also a cardiac activity-related network, and their similar relationships with emotion may suggest that they are the same or a similar kind of network induced by the heartbeat although they were measured by different modalities and methods, with the autonomic network emphasizing a peripheral pulse-cortical region relationship and module 1 of the HIN focusing on heartbeat-induced interactions between cortical regions. However, the regions included in each network were not entirely the same; therefore, we cannot determine whether the automatic network and module 1 are the same in this study. However, we can conclude that a strong relationship exists between the regions included in both networks and heartbeat processing and also between heartbeat-related processing within these regions and the emotional state of an individual.

A limitation of our study is that although interoceptive processing typically includes subcortical regions, such as the amygdala (Kleckner et al., 2017), we used only cortical regions to construct the HIN. The HIN including subcortical regions may show different properties than those reported in this study. Therefore, future studies on HINs including subcortical regions using deep source imaging MEG techniques are needed.

In conclusion, we first showed the existence of HIN interactions with hubs in inferior temporal regions. The HIN was modularized and contained five modules, with module 1 as the center of module interactions. The synchronization within module 1 of the HIN had a positive association with the mood scores of the participants. Considering recent theories on abnormal interoceptive processing in mood disorder patients (Paulus and Stein, 2010), investigating the HIN within such patients may also improve our understanding of the corresponding pathophysiology.

## Synthesis

Reviewing Editor: Daniela Schiller, Mount Sinai School of Medicine Decisions are customarily a result of the Reviewing Editor and the peer reviewers coming together and discussing their recommendations until a consensus is reached. When revisions are invited, a fact-based synthesis statement explaining their decision and outlining what is needed to prepare a revision will be listed below. The following reviewer(s) agreed to reveal their identity: Hugo Critchley. Note: If this manuscript was transferred from JNeurosci and a decision was made to accept the manuscript without peer review, a brief statement to this effect will instead be what is listed below.

The manuscript describes a cardiac couple resting brain networks in the theta frequency band identified from MEG data. This represents a new observation that is potential relevant to embodied cognition and emotion. This relationship and associated information in theta frequency prompt further studies.

The revised analyses are compelling. The authors have addressed the earlier concerns about this paper.

ACTION REQUIRED: Proof reading with respect to grammar and typos would be useful.
