# Supplementary material for: Heartbeat Induces a Cortical Theta-Synchronized Network in the Resting State
Source: eNeuro. 2019 Aug 8;6(4):ENEURO.0200-19.2019. doi: 10.1523/ENEURO.0200-19.2019 (PMC6709221; doi:10.1523/ENEURO.0200-19.2019)
Supplement: Extended Data Table 1-1 — List of 195 cortical regions used in the analysis and the graph theoretical properties of each region Download Table 1-1, DOCX file. [file sup_enu-eN-NWR-0200-19-s02.docx]

**Table 1-1. List of 195 cortical regions used in the analysis and the graph theoretical properties of each region.**

| **Region fullname (Abbreviation, full name)** | **BC** | **Strength** | **Module** |
| --- | --- | --- | --- |
| ITG, Left Inferior Temporal Gyrus A20il, intermediate lateral area 20 | 8661.4 | 30.1 | 5 |
| PhG, Left Parahippocampal Gyrus A35/36r, rostral area 35/36 | 4775.5 | 25.9 | 5 |
| ITG, Left Inferior Temporal Gyrus A20iv, intermediate ventral area 20 | 2781.3 | 25.4 | 4 |
| ITG, Left Inferior Temporal Gyrus A20r, rostral area 20 | 4069.9 | 24.8 | 5 |
| FuG, Left Fusiform Gyrus A20rv, rostroventral area 20 | 1557.0 | 24.3 | 4 |
| OrG, Left Orbital Gyrus A12/47o, orbital area 12/47 | 5621.8 | 21.9 | 1 |
| PhG, Left Parahippocampal Gyrus A28/34, area 28/34 (EC, entorhinal cortex) | 1778.1 | 20.0 | 5 |
| OrG, Right Orbital Gyrus A11m, medial area 11 | 2162.1 | 18.4 | 2 |
| STG, Left Superior Temporal Gyrus A38m, medial area 38 | 3505.0 | 18.0 | 5 |
| MFG, Left Middle Frontal Gyrus A10l, lateral area10 | 2075.6 | 17.2 | 2 |
| OrG, Left Orbital Gyrus A11m, medial area 11 | 1020.2 | 16.7 | 2 |
| MTG, Left Middle Temporal Gyrus A21r, rostral area 21 | 129.7 | 16.4 | 5 |
| PhG, Left Parahippocampal Gyrus A35/36c, caudal area 35/36 | 2718.9 | 16.4 | 1 |
| PhG, Left Parahippocampal Gyrus TI, area TI (temporal agranular insular cortex) | 1219.2 | 15.3 | 5 |
| OrG, Left Orbital Gyrus A12/47l, lateral area 12/47 | 696.6 | 11.8 | 1 |
| INS, Left Insular Gyrus vIa, ventral agranular insula | 9.0 | 9.3 | 5 |
| OrG, Left Orbital Gyrus A11l, lateral area 11 | 101.5 | 8.5 | 1 |
| OrG, Left Orbital Gyrus A14m, medial area 14 | 7.0 | 8.4 | 2 |
| ITG, Left Inferior Temporal Gyrus A20cv, caudoventral of area 20 | 268.4 | 8.4 | 1 |
| STG, Left Superior Temporal Gyrus A38l, lateral area 38 | 1.0 | 8.4 | 5 |
| ITG, Right Inferior Temporal Gyrus A20cv, caudoventral of area 20 | 680.5 | 7.4 | 2 |
| PrG, Left Precentral Gyrus A6cdl, caudal dorsolateral area 6 | 461.4 | 7.3 | 4 |
| PhG, Right Parahippocampal Gyrus A28/34, area 28/34 (EC, entorhinal cortex) | 378.9 | 7.2 | 4 |
| PhG, Left Parahippocampal Gyrus TL, area TL (lateral PPHC, posterior parahippocampal gyrus) | 45.2 | 6.8 | 1 |
| SFG, Left Superior Frontal Gyrus A10m, medial area 10 | 5.0 | 6.7 | 2 |
| FuG, Left Fusiform Gyrus A37lv, lateroventral area37 | 127.6 | 6.7 | 1 |
| ITG, Right Inferior Temporal Gyrus A20cl, caudolateral of area 20 | 758.9 | 6.6 | 2 |
| MVOcC, Left MedioVentral Occipital Cortex rCunG, rostral cuneus gyrus | 200.0 | 6.5 | 5 |
| PCL, Left Paracentral Lobule A4ll, area 4, (lower limb region) | 1.0 | 6.4 | 5 |
| INS, Right Insular Gyrus G, hypergranular insula | 266.4 | 6.4 | 2 |
| PrG, Left Precentral Gyrus A4ul, area 4 (upper limb region) | 282.4 | 6.4 | 4 |
| INS, Left Insular Gyrus dIg, dorsal granular insula | 15.1 | 6.4 | 2 |
| PoG, Left Postcentral Gyrus A1/2/3ulhf, area 1/2/3 (upper limb, head and face region) | 133.7 | 6.4 | 4 |
| MFG, Right Middle Frontal Gyrus A10l, lateral area10 | 7.0 | 6.3 | 5 |
| CG, Left Cingulate Gyrus A23c, caudal area 23 | 190.0 | 6.2 | 5 |
| FuG, Left Fusiform Gyrus A37mv, medioventral area37 | 457.3 | 5.8 | 1 |
| FuG, Right Fusiform Gyrus A20rv, rostroventral area 20 | 175.9 | 5.7 | 2 |
| pSTS, Right posterior Superior Temporal Sulcus rpSTS, rostroposterior superior temporal sulcus | 5.0 | 5.6 | 2 |
| INS, Left Insular Gyrus dIa, dorsal agranular insula | 107.5 | 5.6 | 5 |
| SPL, Left Superior Parietal Lobule A7pc, postcentral area 7 | 181.9 | 5.4 | 1 |
| PhG, Right Parahippocampal Gyrus A35/36c, caudal area 35/36 | 674.4 | 5.3 | 2 |
| Pcun, Left PrecuneusA7m, medial area 7 (PEp) | 95.5 | 5.3 | 5 |
| Pcun, Left PrecuneusA31, area 31 (Lc1) | 256.3 | 5.3 | 5 |
| PoG, Left Postcentral Gyrus A1/2/3tonIa, area 1/2/3 (tongue and larynx region) | 11.0 | 5.2 | 2 |
| SFG, Left Superior Frontal Gyrus A6m, medial area 6 | 364.9 | 5.2 | 5 |
| INS, Right Insular Gyrus dIg, dorsal granular insula | 248.3 | 5.1 | 3 |
| PoG, Left Postcentral Gyrus A2, area 2 | 1.0 | 5.1 | 4 |
| OrG, Right Orbital Gyrus A14m, medial area 14 | 1.0 | 5.0 | 2 |
| STG, Right Superior Temporal Gyrus A38m, medial area 38 | 304.6 | 4.9 | 1 |
| MVOcC, Right MedioVentral Occipital Cortex rLinG, rostral lingual gyrus | 336.7 | 4.8 | 3 |
| PrG, Left Precentral Gyrus A4hf, area 4 (head and face region) | 5.0 | 4.8 | 4 |
| INS, Left Insular Gyrus vId/vIg, ventral dysgranular and granular insula | 1.0 | 4.7 | 2 |
| PoG, Right Postcentral Gyrus A1/2/3tonIa, area 1/2/3 (tongue and larynx region) | 1.0 | 4.6 | 3 |
| ITG, Right Inferior Temporal Gyrus A37vl, ventrolateral area 37 | 7.0 | 4.6 | 4 |
| IPL, Right Inferior Parietal Lobule A40c, caudal area 40 (PFm) | 1.0 | 4.6 | 2 |
| STG, Left Superior Temporal Gyrus A41/42, area 41/42 | 232.2 | 4.6 | 1 |
| MTG, Left Middle Temporal Gyrus aSTS, anterior superior temporal sulcus | 5.0 | 4.5 | 2 |
| PrG, Right Precentral Gyrus A6cdl, caudal dorsolateral area 6 | 1.0 | 4.5 | 5 |
| SFG, Right Superior Frontal Gyrus A6m, medial area 6 | 1.0 | 4.5 | 5 |
| PrG, Left Precentral Gyrus A4t, area 4 (trunk region) | 9.0 | 4.4 | 1 |
| IFG, Right Inferior Frontal Gyrus A44op, opercular area 44 | 141.7 | 4.3 | 3 |
| MTG, Right Middle Temporal Gyrus A37dl, dorsolateral area37 | 350.8 | 4.3 | 5 |
| PCL, Right Paracentral Lobule A4ll, area 4, (lower limb region) | 1.0 | 4.2 | 5 |
| STG, Right Superior Temporal Gyrus A38l, lateral area 38 | 630.2 | 4.1 | 3 |
| CG, Right Cingulate Gyrus A23c, caudal area 23 | 161.8 | 4.1 | 5 |
| CG, Left Cingulate Gyrus A24rv, rostroventral area 24 | 326.7 | 4.1 | 3 |
| PoG, Left Postcentral Gyrus A1/2/3tru, area1/2/3 (trunk region) | 67.3 | 4.1 | 1 |
| IFG, Left Inferior Frontal Gyrus A45r, rostral area 45 | 3.0 | 4.1 | 2 |
| PCL, Left Paracentral Lobule A1/2/3ll, area 1/2/3 (lower limb region) | 1.0 | 4.0 | 5 |
| IFG, Left Inferior Frontal Gyrus A44op, opercular area 44 | 1.0 | 4.0 | 5 |
| CG, Right Cingulate Gyrus A32p, pregenual area 32 | 1.0 | 3.9 | 1 |
| PrG, Right Precentral Gyrus A6cvl, caudal ventrolateral area 6 | 1.0 | 3.8 | 2 |
| Pcun, Left PrecuneusA5m, medial area 5 (PEm) | 1.0 | 3.8 | 5 |
| IPL, Right Inferior Parietal Lobule A40rv, rostroventral area 40 (PFop) | 1.0 | 3.8 | 2 |
| ITG, Right Inferior Temporal Gyrus A20iv, intermediate ventral area 20 | 119.6 | 3.8 | 2 |
| SFG, Left Superior Frontal Gyrus A8m, medial area 8 | 1.0 | 3.7 | 2 |
| INS, Left Insular Gyrus G, hypergranular insula | 147.7 | 3.7 | 4 |
| IPL, Right Inferior Parietal Lobule A40rd, rostrodorsal area 40 (PFt) | 1.0 | 3.7 | 5 |
| Pcun, Right PrecuneusA7m, medial area 7 (PEp) | 1.0 | 3.7 | 5 |
| PhG, Right Parahippocampal Gyrus TL, area TL (lateral PPHC, posterior parahippocampal gyrus) | 282.4 | 3.6 | 2 |
| FuG, Right Fusiform Gyrus A37lv, lateroventral area37 | 1.0 | 3.6 | 2 |
| CG, Left Cingulate Gyrus A23v, ventral area 23 | 1.0 | 3.6 | 5 |
| SFG, Right Superior Frontal Gyrus A9l, lateral area 9 | 1.0 | 3.4 | 4 |
| SPL, Left Superior Parietal Lobule A5l, lateral area 5 | 137.7 | 3.4 | 1 |
| IFG, Right Inferior Frontal Gyrus IFS, inferior frontal sulcus | 31.1 | 3.4 | 3 |
| STG, Left Superior Temporal Gyrus TE1.0 and TE1.2 | 35.2 | 3.3 | 2 |
| IFG, Left Inferior Frontal Gyrus A45c, caudal area 45 | 1.0 | 3.3 | 4 |
| SFG, Right Superior Frontal Gyrus A10m, medial area 10 | 1.0 | 3.3 | 1 |
| CG, Left Cingulate Gyrus A24cd, caudodorsal area 24 | 348.8 | 3.3 | 4 |
| MFG, Right Middle Frontal Gyrus A6vl, ventrolateral area 6 | 1.0 | 3.2 | 1 |
| ITG, Left Inferior Temporal Gyrus A20cl, caudolateral of area 20 | 7.0 | 3.2 | 1 |
| LOcC, Left lateral Occipital Cortex lsOccG, lateral superior occipital gyrus | 1.0 | 3.2 | 5 |
| SFG, Right Superior Frontal Gyrus A9m, medial area 9 | 1.0 | 3.2 | 4 |
| CG, Left Cingulate Gyrus A32sg, subgenual area 32 | 1.0 | 3.0 | 2 |
| MVOcC, Left MedioVentral Occipital Cortex rLinG, rostral lingual gyrus | 292.5 | 3.0 | 1 |
| SPL, Left Superior Parietal Lobule A7c, caudal area 7 | 5.0 | 3.0 | 5 |
| IPL, Right Inferior Parietal Lobule A39rv, rostroventral area 39 (PGa) | 1.0 | 2.9 | 4 |
| INS, Right Insular Gyrus dIa, dorsal agranular insula | 7.0 | 2.9 | 3 |
| IPL, Left Inferior Parietal Lobule A40rd, rostrodorsal area 40 (PFt) | 47.2 | 2.9 | 4 |
| OrG, Right Orbital Gyrus A13, area 13 | 17.1 | 2.9 | 4 |
| MVOcC, Right MedioVentral Occipital Cortex vmPOS, ventromedial parietooccipital sulcus | 1.0 | 2.9 | 4 |
| STG, Right Superior Temporal Gyrus A41/42, area 41/42 | 1.0 | 2.9 | 4 |
| Pcun, Right PrecuneusA5m, medial area 5 (PEm) | 1.0 | 2.9 | 5 |
| SFG, Left Superior Frontal Gyrus A8dl, dorsolateral area 8 | 5.0 | 2.9 | 2 |
| MTG, Right Middle Temporal Gyrus A21r, rostral area 21 | 3.0 | 2.9 | 3 |
| CG, Right Cingulate Gyrus A32sg, subgenual area 32 | 1.0 | 2.8 | 2 |
| INS, Right Insular Gyrus vIa, ventral agranular insula | 47.2 | 2.7 | 3 |
| IPL, Left Inferior Parietal Lobule A39rd, rostrodorsal area 39 (Hip3) | 1.0 | 2.7 | 3 |
| CG, Left Cingulate Gyrus A23d, dorsal area 23 | 1.0 | 2.7 | 5 |
| SPL, Left Superior Parietal Lobule A7r, rostral area 7 | 1.0 | 2.6 | 5 |
| Pcun, Right PrecuneusA31, area 31 (Lc1) | 1.0 | 2.6 | 5 |
| PhG, Left Parahippocampal Gyrus TH, area TH (medial PPHC) | 11.0 | 2.6 | 3 |
| SFG, Right Superior Frontal Gyrus A6dl, dorsolateral area 6 | 1.0 | 2.5 | 4 |
| CG, Left Cingulate Gyrus A32p, pregenual area 32 | 1.0 | 2.5 | 1 |
| CG, Right Cingulate Gyrus A24rv, rostroventral area 24 | 3.0 | 2.4 | 1 |
| ITG, Left Inferior Temporal Gyrus A37elv, extreme lateroventral area37 | 77.4 | 2.4 | 1 |
| ITG, Right Inferior Temporal Gyrus A37elv, extreme lateroventral area37 | 5.0 | 2.4 | 1 |
| MFG, Left Middle Frontal Gyrus A9/46d, dorsal area 9/46 | 71.4 | 2.4 | 5 |
| INS, Left Insular Gyrus dId, dorsal dysgranular insula | 1.0 | 2.4 | 2 |
| PrG, Right Precentral Gyrus A4hf, area 4 (head and face region) | 1.0 | 2.2 | 4 |
| MFG, Left Middle Frontal Gyrus A6vl, ventrolateral area 6 | 1.0 | 2.2 | 4 |
| SFG, Right Superior Frontal Gyrus A8dl, dorsolateral area 8 | 13.1 | 2.2 | 3 |
| STG, Left Superior Temporal Gyrus A22r, rostral area 22 | 1.0 | 2.2 | 2 |
| INS, Right Insular Gyrus vId/vIg, ventral dysgranular and granular insula | 139.7 | 2.2 | 3 |
| PrG, Right Precentral Gyrus A4tl, area 4 (tongue and larynx region) | 5.0 | 2.1 | 3 |
| SFG, Left Superior Frontal Gyrus A9m, medial area 9 | 1.0 | 2.1 | 1 |
| IPL, Left Inferior Parietal Lobule A40c, caudal area 40 (PFm) | 1.0 | 2.1 | 3 |
| MFG, Right Middle Frontal Gyrus A46, area 46 | 137.7 | 2.1 | 5 |
| SFG, Left Superior Frontal Gyrus A6dl, dorsolateral area 6 | 7.0 | 2.1 | 1 |
| Pcun, Left PrecuneusdmPOS, dorsomedial parietooccipital sulcus (PEr) | 3.0 | 2.1 | 1 |
| SPL, Right Superior Parietal Lobule A5l, lateral area 5 | 1.0 | 2.1 | 5 |
| MTG, Left Middle Temporal Gyrus A21c, caudal area 21 | 33.2 | 2.0 | 1 |
| pSTS, Right posterior Superior Temporal Sulcus cpSTS, caudoposterior superior temporal sulcus | 1.0 | 2.0 | 4 |
| PCL, Right Paracentral Lobule A1/2/3ll, area 1/2/3 (lower limb region) | 1.0 | 2.0 | 5 |
| ITG, Right Inferior Temporal Gyrus A20il, intermediate lateral area 20 | 5.0 | 2.0 | 2 |
| OrG, Right Orbital Gyrus A11l, lateral area 11 | 9.0 | 2.0 | 2 |
| OrG, Right Orbital Gyrus A12/47l, lateral area 12/47 | 31.1 | 1.9 | 3 |
| IFG, Left Inferior Frontal Gyrus IFS, inferior frontal sulcus | 1.0 | 1.9 | 2 |
| FuG, Right Fusiform Gyrus A37mv, medioventral area37 | 47.2 | 1.9 | 3 |
| IPL, Left Inferior Parietal Lobule A39c, caudal area 39 (PGp) | 1.0 | 1.9 | 5 |
| PoG, Right Postcentral Gyrus A2, area 2 | 1.0 | 1.9 | 2 |
| PrG, Right Precentral Gyrus A4t, area 4 (trunk region) | 1.0 | 1.9 | 1 |
| MFG, Left Middle Frontal Gyrus IFJ, inferior frontal junction | 1.0 | 1.8 | 2 |
| MFG, Left Middle Frontal Gyrus A9/46v, ventral area 9/46 | 1.0 | 1.8 | 2 |
| SPL, Left Superior Parietal Lobule A7ip, intraparietal area 7 (hIP3) | 1.0 | 1.8 | 1 |
| SPL, Right Superior Parietal Lobule A7r, rostral area 7 | 1.0 | 1.8 | 5 |
| OrG, Right Orbital Gyrus A12/47o, orbital area 12/47 | 43.2 | 1.7 | 1 |
| STG, Right Superior Temporal Gyrus TE1.0 and TE1.2 | 1.0 | 1.7 | 2 |
| SPL, Right Superior Parietal Lobule A7c, caudal area 7 | 1.0 | 1.7 | 5 |
| IFG, Left Inferior Frontal Gyrus A44d, dorsal area 44 | 7.0 | 1.7 | 2 |
| SFG, Left Superior Frontal Gyrus A9l, lateral area 9 | 1.0 | 1.7 | 4 |
| MTG, Right Middle Temporal Gyrus aSTS, anterior superior temporal sulcus | 1.0 | 1.6 | 3 |
| OrG, Left Orbital Gyrus A13, area 13 | 3.0 | 1.6 | 5 |
| CG, Right Cingulate Gyrus A24cd, caudodorsal area 24 | 1.0 | 1.5 | 4 |
| MFG, Left Middle Frontal Gyrus A46, area 46 | 7.0 | 1.5 | 5 |
| PrG, Left Precentral Gyrus A6cvl, caudal ventrolateral area 6 | 1.0 | 1.5 | 2 |
| IFG, Right Inferior Frontal Gyrus A45r, rostral area 45 | 9.0 | 1.5 | 4 |
| PoG, Right Postcentral Gyrus A1/2/3ulhf, area 1/2/3 (upper limb, head and face region) | 5.0 | 1.5 | 4 |
| MFG, Left Middle Frontal Gyrus A8vl, ventrolateral area 8 | 1.0 | 1.4 | 1 |
| IFG, Right Inferior Frontal Gyrus A44d, dorsal area 44 | 1.0 | 1.4 | 3 |
| CG, Right Cingulate Gyrus A23d, dorsal area 23 | 1.0 | 1.3 | 5 |
| INS, Right Insular Gyrus dId, dorsal dysgranular insula | 7.0 | 1.3 | 3 |
| MFG, Right Middle Frontal Gyrus A8vl, ventrolateral area 8 | 1.0 | 1.3 | 1 |
| IFG, Right Inferior Frontal Gyrus A44v, ventral area 44 | 35.2 | 1.2 | 3 |
| MFG, Right Middle Frontal Gyrus IFJ, inferior frontal junction | 248.3 | 1.2 | 1 |
| STG, Right Superior Temporal Gyrus A22r, rostral area 22 | 1.0 | 1.2 | 3 |
| pSTS, Left posterior Superior Temporal Sulcus rpSTS, rostroposterior superior temporal sulcus | 9.0 | 1.2 | 1 |
| SFG, Right Superior Frontal Gyrus A8m, medial area 8 | 1.0 | 1.2 | 1 |
| MFG, Right Middle Frontal Gyrus A9/46v, ventral area 9/46 | 1.0 | 1.2 | 2 |
| PrG, Left Precentral Gyrus A4tl, area 4 (tongue and larynx region) | 1.0 | 1.1 | 2 |
| IFG, Left Inferior Frontal Gyrus A44v, ventral area 44 | 1.0 | 1.1 | 4 |
| LOcC, Right lateral Occipital Cortex lsOccG, lateral superior occipital gyrus | 1.0 | 1.1 | 5 |
| MFG, Right Middle Frontal Gyrus A9/46d, dorsal area 9/46 | 1.0 | 1.1 | 5 |
| Pcun, Right PrecuneusdmPOS, dorsomedial parietooccipital sulcus (PEr) | 1.0 | 1.1 | 1 |
| PhG, Right Parahippocampal Gyrus TI, area TI (temporal agranular insular cortex) | 1.0 | 1.1 | 4 |
| IFG, Right Inferior Frontal Gyrus A45c, caudal area 45 | 1.0 | 1.0 | 3 |
| STG, Left Superior Temporal Gyrus A22c, caudal area 22 | 1.0 | 1.0 | 2 |
| MTG, Right Middle Temporal Gyrus A21c, caudal area 21 | 350.8 | 1.0 | 3 |
| IPL, Left Inferior Parietal Lobule A40rv, rostroventral area 40 (PFop) | 3.0 | 1.0 | 1 |
| PhG, Right Parahippocampal Gyrus TH, area TH (medial PPHC) | 3.0 | 1.0 | 2 |
| LOcC, Right lateral Occipital Cortex V5/MT+, area V5/MT+ | 1.0 | 1.0 | 4 |
| STG, Right Superior Temporal Gyrus A22c, caudal area 22 | 1.0 | 0.9 | 2 |
| ITG, Left Inferior Temporal Gyrus A37vl, ventrolateral area 37 | 1.0 | 0.8 | 1 |
| MVOcC, Left MedioVentral Occipital Cortex vmPOS, ventromedial parietooccipital sulcus | 1.0 | 0.8 | 1 |
| MTG, Left Middle Temporal Gyrus A37dl, dorsolateral area37 | 1.0 | 0.8 | 5 |
| IPL, Left Inferior Parietal Lobule A39rv, rostroventral area 39 (PGa) | 1.0 | 0.7 | 1 |
| IPL, Right Inferior Parietal Lobule A39c, caudal area 39 (PGp) | 1.0 | 0.6 | 1 |
| PrG, Right Precentral Gyrus A4ul, area 4 (upper limb region) | 1.0 | 0.4 | 1 |
| SPL, Right Superior Parietal Lobule A7ip, intraparietal area 7 (hIP3) | 1.0 | 0.4 | 5 |
| SPL, Right Superior Parietal Lobule A7pc, postcentral area 7 | 1.0 | 0.4 | 4 |
| CG, Right Cingulate Gyrus A23v, ventral area 23 | 3.0 | 0.3 | 3 |
| LOcC, Left lateral Occipital Cortex V5/MT+, area V5/MT+ | 1.0 | 0.3 | 1 |
| IPL, Right Inferior Parietal Lobule A39rd, rostrodorsal area 39 (Hip3) | 1.0 | 0.2 | 1 |
| PoG, Right Postcentral Gyrus A1/2/3tru, area1/2/3 (trunk region) | 1.0 | 0.2 | 1 |
| pSTS, Left posterior Superior Temporal Sulcus cpSTS, caudoposterior superior temporal sulcus | 0.0 | 0.0 | no module assigned |

**BC: Betweenness centrality**
