# Supplementary figures and images for: Heartbeat Induces a Cortical Theta-Synchronized Network in the Resting State
Source: eNeuro. 2019 Aug 8;6(4):ENEURO.0200-19.2019. doi: 10.1523/ENEURO.0200-19.2019 (PMC6709221; doi:10.1523/ENEURO.0200-19.2019)

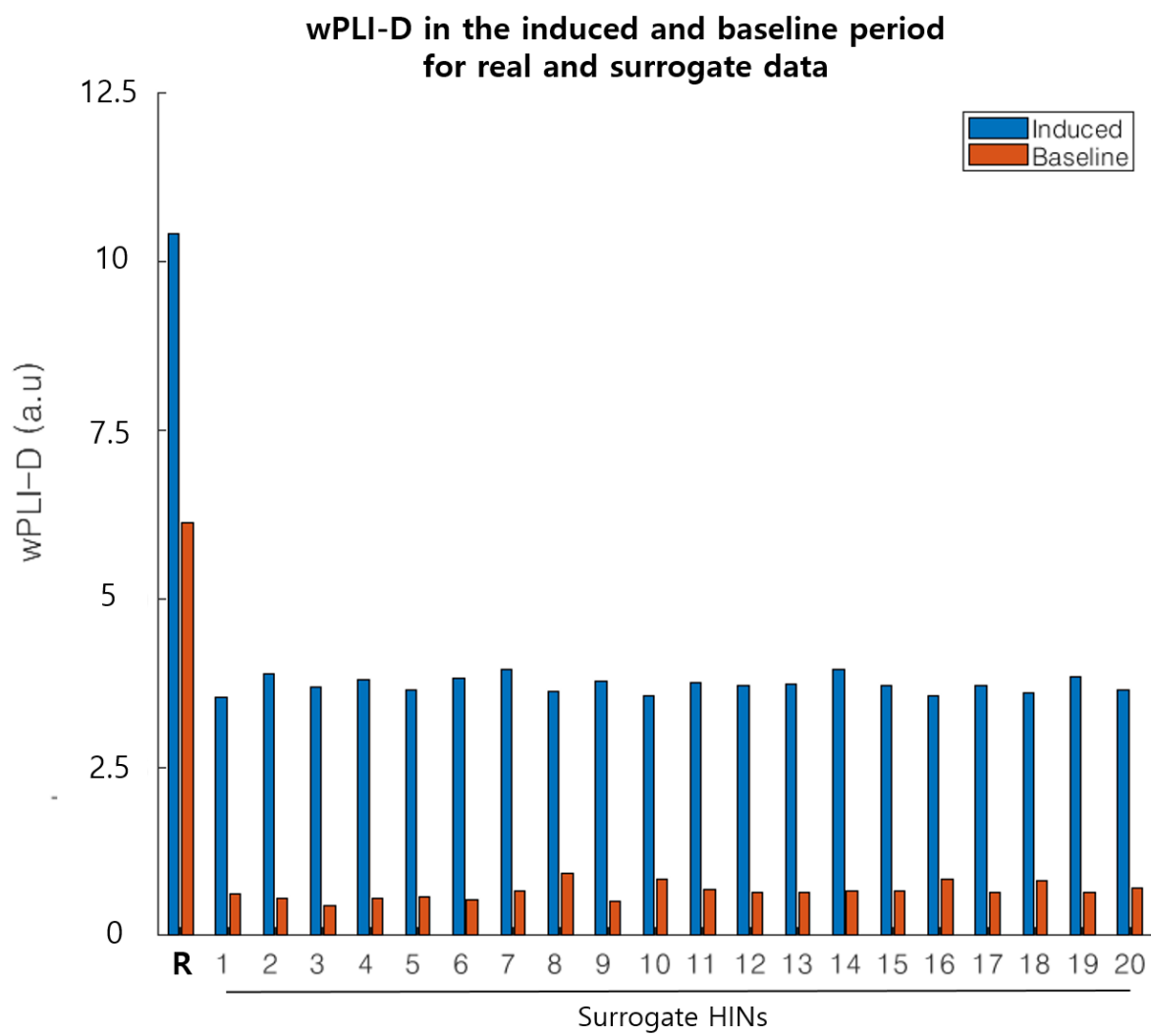

**Figure 1-1.**

Supplement: Extended Data Figure 1-1 — wPLI-D in the induced and baseline period for real and surrogate data. This figure shows the wPLI-D in the induced (blue) and baseline (orange) period separately. R represents the wPLI-D of the real data and others represents wPLI-D of 20 surrogate data. One can notice that the synchronization within the HIN is much stronger in the real data for both induced and baseline period. Note that, an increase of induced synchronization was also strongest in the real data compared to the surrogate data (Fig. 1B). Download Figure 1-1, PDF file. [file sup_enu-eN-NWR-0200-19-s01.pdf]
